# Effect of modified Total Body Recumbent Stepper training on exercise capacity and thioredoxin in COPD: a randomized clinical trial

**DOI:** 10.1038/s41598-022-15466-2

**Published:** 2022-07-01

**Authors:** Wentao Duan, Dan Zeng, Jin Huang, Jing Gu, San Li, Wei Zhou, Jinling Ma, Yan Jiang, Liming Zhu, Xudong Xiang, Aiguo Dai

**Affiliations:** 1 Department of Respiratory and Critical Care Medicine, Research Unit of Respiratory Diseases, The Second Xiangya Hospital of Central South University, Changsha, 410011 China; 2 Department of Respiratory and Critical Care Medicine, Hunan Provincial People’s Hospital (The First-Affiliated Hospital of Hunan Normal University), 410016 Changsha, China; 3 Institute of Respiratory Disease, Hunan Provincial People’s Hospital (The First-Affiliated Hospital of Hunan Normal University), Changsha, 410016 China; 4 Department of Emergency, Institute of Emergency and Difficult Diseases, The Second Xiangya Hospital of Central South University, Changsha, 410011 China; 5 Department of Respiratory Diseases, Medical School, Hunan University of Chinese Medicine/Key Laboratory of Vascular Biology and Translational Medicine in Hunan Province, Changsha, 410208 China

**Keywords:** Chronic obstructive pulmonary disease, Rehabilitation, Quality of life

## Abstract

Exercise intolerance is one of the major symptoms of chronic obstructive pulmonary disease (COPD). Exercise training can benefit COPD patients, but the underlying mechanism remains unclear. The modified Total Body Recumbent Stepper (TBRS, Nustep-T4) can benefit patients with stroke, spinal cord injury and amyotrophic lateral sclerosis. Nevertheless, the effect of TBRS training alone on pulmonary rehabilitation (PR) in COPD patients remains largely unknown. We aimed to explore the effect of TBRS training on exercise capacity and the thioredoxin system (TRXS) in COPD patients to provide a novel rehabilitation modality and new theoretical basis for PR of COPD patients. Ninety stable COPD patients were randomly divided into a control group (NC group) and a TBRS training group (TBRS group), with 45 cases in each group. Subjects in the TBRS training group were scheduled to undergo TBRS endurance training triweekly for 12 weeks under the guidance of a rehabilitation therapist. We assessed the primary outcome: exercise capacity (6-min walking distance, 6MWD); and secondary outcomes: perception of dyspnoea (mMRC, Borg), the COPD assessment test (CAT), the BODE index, pulmonary function, the number of acute exacerbations of COPD and oxidative stress (TRXS) at one-year follow-up. Compared with before the intervention and the control group, after the intervention, the TBRS training group, exhibited an increase in the 6MWD (from 366.92 ± 85.81 to 484.10 ± 71.90, 484.10 ± 71.90 vs 370.63 ± 79.87, *P* < 0.01), while the scores on the BORG, mMRC, BODE index, CAT, and the number of acute exacerbations of COPD were reduced, and the protein and mRNA expression levels of TRXS was significantly increased (*P* < 0.01). However, no differences were found in PF parameters in the comparison with before the intervention or between groups. TBRS training can effectively increase exercise capacity, while there are indications that it can alleviate COPD-related dyspnoea and reduce the number of acute exacerbations of COPD. Interestingly, long-term regular TBRS training may reduce oxidative stress associated with COPD to increase exercise capacity.

## Introduction

Chronic obstructive pulmonary disease (COPD) is a preventable and treatable disease characterized by persistent airflow restriction, which is usually progressive and seriously affects exercise ability and quality of life^1^. Most COPD patients have reduced exercise capacity, which seriously affects their quality of life. Pulmonary rehabilitation (PR) is one of the main management strategies for stable COPD and has been recommended by the Global Initiative on COPD as the primary method of nonpharmacological treatment. Moreover, exercise training is considered the cornerstone of PR, and can improve exercise capacity and physical symptoms and significantly improve quality of life in COPD patients^2^. However, due to exercise intolerance, many COPD patients are often unable to achieve the target intensity or duration of exercise prescription to obtain the benefits, which is especially true in for individuals with moderate or severe COPD. Total Body Recumbent Stepper (TBRS) training can benefit patients with stroke, spinal cord injury and amyotrophic lateral sclerosis^3–5^.

Nevertheless, the effect of TBRS training alone on pulmonary rehabilitation in COPD patients remains largely unknown. This paper explored the effect of TBRS training on exercise capacity and thioredoxin system (TRXS) in stable COPD patients and provides a novel rehabilitation modality and theoretical basis for pulmonary rehabilitation in moderate and severe COPD patients.

Upper and lower limb exercises are equally important. Upper limb exercise training can increase forearm motility, reduce the need for ventilation and improve upper limb exercise endurance. TBRS training simulates normal body movement in daily life, in which the upper limbs are flexed, and the lower limbs are in pedal movement mode, so that the upper and lower limbs can obtain active or passive exercise training at the same time, and the movement resistance and duration can be set to attain the target heart rate. TBRS training is an alternative exercise modality commonly used in fitness and rehabilitation settings. Due to the prevalence of low physical activity in COPD patients, it is essential to promote regular physical activity in this segment of the population, especially for those with moderate and severe COPD with severe physical symptoms.

Oxidative stress is an important feature of the pathogenesis of COPD. Many studies have shown that compared with healthy people, COPD patients have increased pulmonary oxidative stress^6–11^. TRXS is one of the major antioxidant systems in the body. However, the mechanism by which TRXS is involved in the pathogenesis of COPD is still not clear. TRXS, composed of thioredoxin (TRX) and thioredoxin reductase (TRXR), plays a key role in the regulation of redox balance and cell proliferation. TRXS is considered a biomarker of oxidative stress^12^ and plays an antioxidative stress role in cells^13^. Redox imbalance results in tissue damage and systemic inflammation in COPD patients, while exercise training can effectively reduce oxidative stress in the body and increase exercise tolerance^14^. Aiming at the target of antioxidant therapy for COPD, exercise training may promote the expression of the endogenous antioxidant TRXS in the body and have an effect on the treatment and rehabilitation of COPD patients.

## Methods

### Study design

All recruited COPD patients were followed up in the Department of Respiratory Medicine and the Department of Elderly Respiratory Medicine of Hunan Provincial People's Hospital from June 2016 to April 2020. This study was approved by the Ethics Committee of Hunan Provincial People's Hospital (No. 2015020-1), all methods performed were in accordance with the 1964 Helsinki declaration and its later amendments or comparable ethical standards. The study was retrospectively registered at the Chinese Clinical Trial Registry on 23/10/2021 (No. ChiCTR2100052230). All the enrolled patients were informed of the relevant details before the study, and all participants signed an informed consent form including publication of identifying information/images in an online open-access publication. Each patient’s contact information, address, age, past history, pulmonary function test (PFT), pulmonary rehabilitation measurements and number of acute exacerbations of COPD before and after a year were collected. Reporting of this trial was consistent with the Consolidated Standard of Reporting Trials statement^15^.

### Recruitment of subjects

Of the 100 COPD subjects initially enrolled, 90 eligible COPD patients who met the inclusion criteria were included in the clinical trial. These patients were randomly assigned to the control group (NC group) or the TBRS training group (TBRS group) according to a computer-generated sequence using a simple randomization method. The randomization list was concealed in sequentially numbered, sealed, opaque envelopes and prepared by an independent physician not involved in subject recruitment. Each new subject was assigned a sequential number, and then the corresponding envelope was opened to decide which group they would enter.

The inclusion criteria were (a) COPD stage II-III and (b) maintenance of stable COPD condition for more than 4 weeks according to the Chronic Obstructive Lung Disease guidelines (GOLD)^16^.

The exclusion criteria were (a) contraindications to exercise training; (b) conditions that might affect the pulmonary oxidative stress response^17,18^: serious liver, kidney and endocrine diseases; diseases of the nervous system; serious cardiovascular and cerebrovascular diseases; connective tissue diseases; glaucoma and (c) antioxidant treatment.

### Study interventions

**Figure 1:**
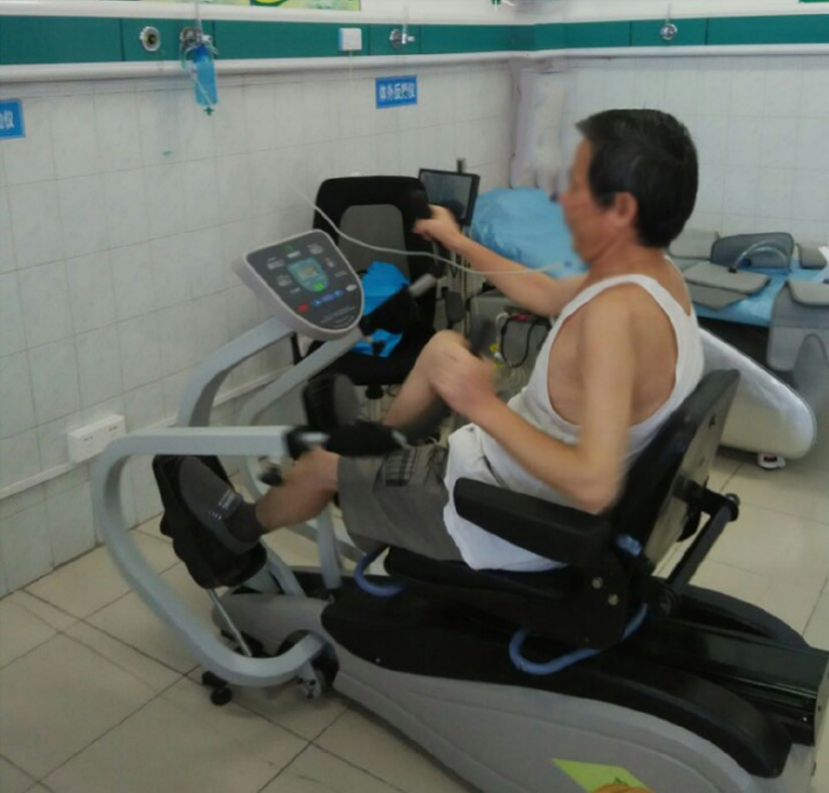
COPD volunteers was performing exercise training on the TBRS while inhaling oxygen.

All the enrolled COPD patients maintained stable COPD drug therapy according to GOLD^16^. Participants in the NC group kept their daily activities as usual and were followed up by telephone. The COPD patients in the TBRS group performed exercise training on a TBRS (NuStep-T4, America) under the guidance of a rehabilitation therapist in the pulmonary rehabilitation room. TBRS could synchronously display the patient's pace frequency, pace speed, accumulated steps, heart rate and heat, and the resistance of the instrument could be adjusted from grade 1 to 10 according to the patient's situation. Heart rate reserve (HRR) was used to evaluate the intensity of exercise training^19^, target heart rate (THR) was calculated as (HRmax − HRrest) × expected intensity % + HRrest, HRmax was 220—age and the expected intensity was initially 60% and increased gradually according to the patient's adaptive intensity^20^. Participants in the TBRS group were advised to speed up at a lower grade load initially and/or increase the load to achieve higher exercise intensity under the guidance of a rehabilitation therapist, and the duration of the exercise protocol was 12 weeks, with three 30-min sessions per week. Blood oxygen saturation and heart rate were monitored, and COPD patients were required to inhale oxygen if their oxygen saturation fell below 88% (Fig. 1). We performed two assessments (in the TBRS group at enrolment and after 12 weeks of TBRS training intervention, and in the NC group at enrolment and 12 weeks later) for all enrolled COPD patients.

The drop-out criteria were (a) death, (b) acute exacerbation of COPD according to GOLD, (c) withdrawal from the study for objective reasons and (d) inability to exercise due to a sudden external condition.

### Primary objective assessment

#### Exercise capacity assessment

Before and after the intervention, exercise capacity was evaluated for all enrolled COPD patients according to the 6-min walking distance (6MWD), which was conducted for COPD patients according to the “6-min walking test guideline” formulated by the American Thoracic Society (ATS)^21^.

### Secondary objective assessment

#### Physical symptoms and COPD assessment

Before and after the intervention, dyspnoea was assessed using the Modified British Medical Research Council (mMRC) Respiration Questionnaire^22^ and the Borg Scale^23^. The COPD Self-Assessment Test (CAT)^24^ and the BODE index^25^ (body mass index, airflow obstruction, dyspnoea, and exercise capacity index) were used for comprehensive assessment of COPD.

#### Measurement of pulmonary function

Before and after the intervention, all enrolled COPD patients were assessed for pulmonary function (JAEGER master screen body plethymograph, Germany)^26^. Then, we chose FEV_1_% pred and FEV_1_/FVC for statistical analysis.

#### Measurement of oxidative stress

Before and after the intervention, patients were asked to avoid moderate- and high-intensity exercise for 2 h. A 10 ml volume of peripheral blood of included patients was divided into two EDTA anticoagulant tubes. The protein and mRNA expression of TRX and TRXR were examined by ELISA (CUSABIO, China) and real-time PCR, respectively.

### Statistical analysis

The power calculation for exercise capacity (6MWD) was performed according to previous studies of pulmonary rehabilitation exercise training for COPD intervention^27,28^. We conservatively estimated the standard deviation to be 18 m (twice as high as reported) in 6MWD and proposed a sample size of 45 patients in each group (alpha = 0.05, power = 0.9 and assuming a loss percentage of 15%).

SPSS Statistics v. 26.0 (IBM Corp., Armonk, NY, USA) was used for statistical analysis. In this study, comparison of test results before and after the intervention was performed using paired or repeated-measures Student’s t tests, and the data were expressed as the mean ± variance (x ± s) when the measurement data conformed to the normal distribution and met the assumption of homogeneity of variance; otherwise, the Wilcoxon test was applied and the data were represented as the median and interquartile range (M [P25–P75]). Comparisons between groups were performed using one-way analysis of variance (ANOVA) or the Welch test where appropriate, and for data that did not follow a normal distribution, we applied the Kruskal–Wallis test. A *P* value < 0.05 indicated a statistically significant difference.

### Ethics statement and consent to participate

This study was approved by the Ethics Review Committee of the Hunan Provincial People's Hospital and all participants signed informed consent (No. 2015020-1).

## Results

**Figure 2:**
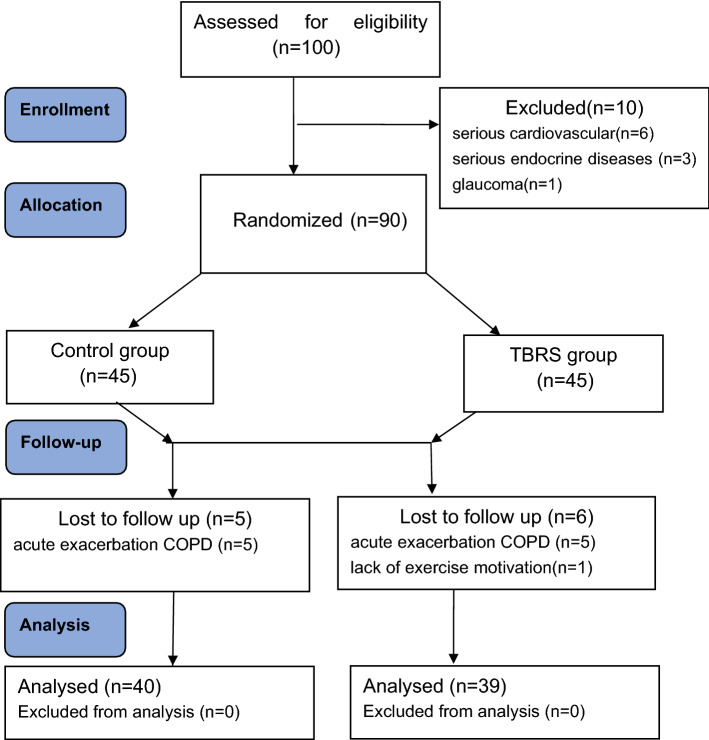
Flow diagram of the study.

One hundred patients with moderate to severe obstruction in a stable phase of COPD were assessed for eligibility, but only 90 patients were enrolled. In the NC group, five COPD patients dropped out (five for acute exacerbation of COPD), and in the TBRS group, six COPD patients dropped out (five for acute exacerbation of COPD and one for lack of exercise motivation). A flow diagram detailing the recruitment of patients can be found in Fig. 2. Accordingly, the analysis was based on 40 patients in the NC group and 39 patients in the TBRS group. The baseline characteristics of the COPD patients who constituted the study population are presented in Table 1. All patients were of Asian descent, strictly met the inclusion and exclusion criteria and were being treated according to the GOLD standard of COPD treatment. Patients had moderate to severe airflow obstruction. No differences in age, sex distribution or pulmonary function were found in patients belonging to the two study groups.

**Table 1:** The main data at baseline (before the interventions).

Items	NC group (n = 45)	TBRS group (n = 45)	*P* value
Age (years), M ± SD	72.67 ± 7.21	72.27 ± 7.16	0.79
FEV_1_%pred, M ± SD	40.57 ± 13.64	43.66 ± 14.76	0.30
FEV_1_/FVC, M ± SD	43.82 ± 9.98	46.37 ± 10.56	0.24
6MWD (m), M ± SD	375.36 ± 72.92	367.11 ± 84.83	0.62
mMRC, median (IQR)	3.0 (2.0–3.0)	3.0 (2.0–3.0)	0.52
Borg, M ± SD	5.24 ± 1.92	4.96 ± 1.78	0.46
CAT, M ± SD	23.87 ± 4.28	22.33 ± 4.51	0.10
BODE, median (IQR)	5.0 (4.00–7.00)	4.0 (3.00–6.00)	0.13
Smoking index, M ± SD	880.53 ± 570.57	914.71 ± 681.37	0.80
BMI, median (IQR)	21.64 ± 1.81	22.11 ± 1.50	0.19

FEV1, forced expiratory volume in 1 s; FEV1%pred, forced expiratory volume in 1 s% prediction; FVC, forced vital capacity; 6MWD, 6-min walk distance; mMRC, modified British medical research council dyspnea questionnaire; Borg, Borg dyspnea score; CAT, COPD assessment test; BODE, body mass index, airflow obstruction, dyspnea, and exercise capacity index; BMI, Body mass index; Smoking index, number of cigarettes smoked per day _*_ number of years smoked. M ± SD, mean ± standard deviation; IQR, interquartile range.

### Primary outcome

#### Exercise capacity assessment

**Figure 3:**
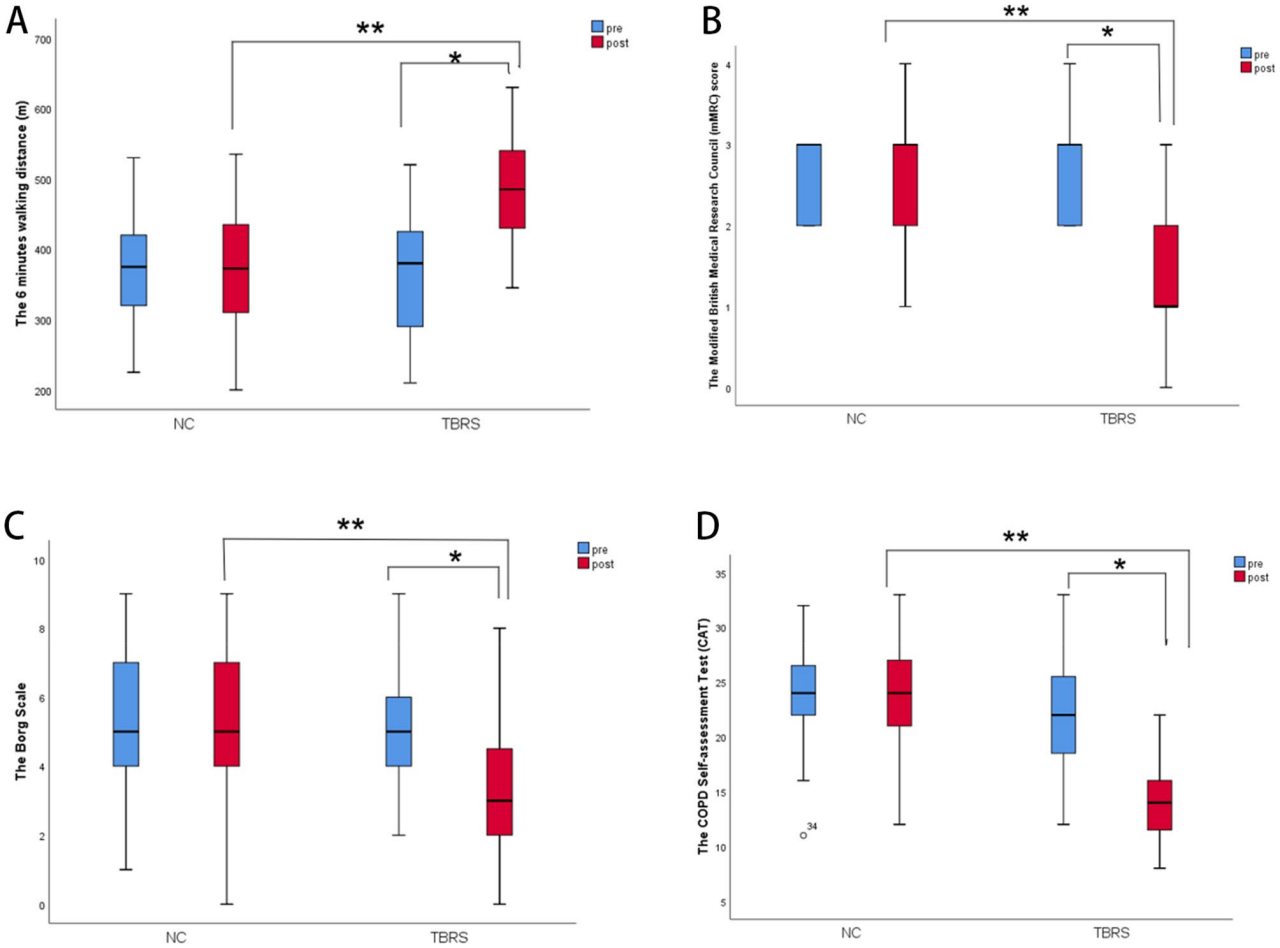
Measurement of pulmonary rehabilitation in the two groups before and after intervention. (**A**) The 6 min walk distance (6MWD); (**B**) the Modified British Medical Research Council (mMRC); (**C**) the Borg scale; (**D**) the COPD Self-assessment Test (CAT); (*P = 0.00 compared with before intervention, **P = 0.00 compared with the control group).

After 12 weeks of TBRS training, exercise capacity improved in the TBRS group but not in the NC group. Compared with before intervention, the 6MWD was significantly increased in the TBRS group (from 366.92 ± 85.81 to 484.10 ± 71.90, *P* = 0.00). Compared with the NC group, the 6MWD was also increased in the TBRS group (484.10 ± 71.90 versus 370.63 ± 79.87, *P* = 0.00) (Fig. 3A).

### Secondary outcomes

#### Dyspnoea assessments

After the intervention, compared with before intervention, the mMRC and Borg scale scores were reduced in the TBRS group (*P* = 0.00). Compared with the NC group, the mMRC (Fig. 3B) and Borg scale (Fig. 3C) scores were also reduced in the TBRS group (*P* = 0.00).

#### The CAT score and BODE index assessment

**Figure 4:**
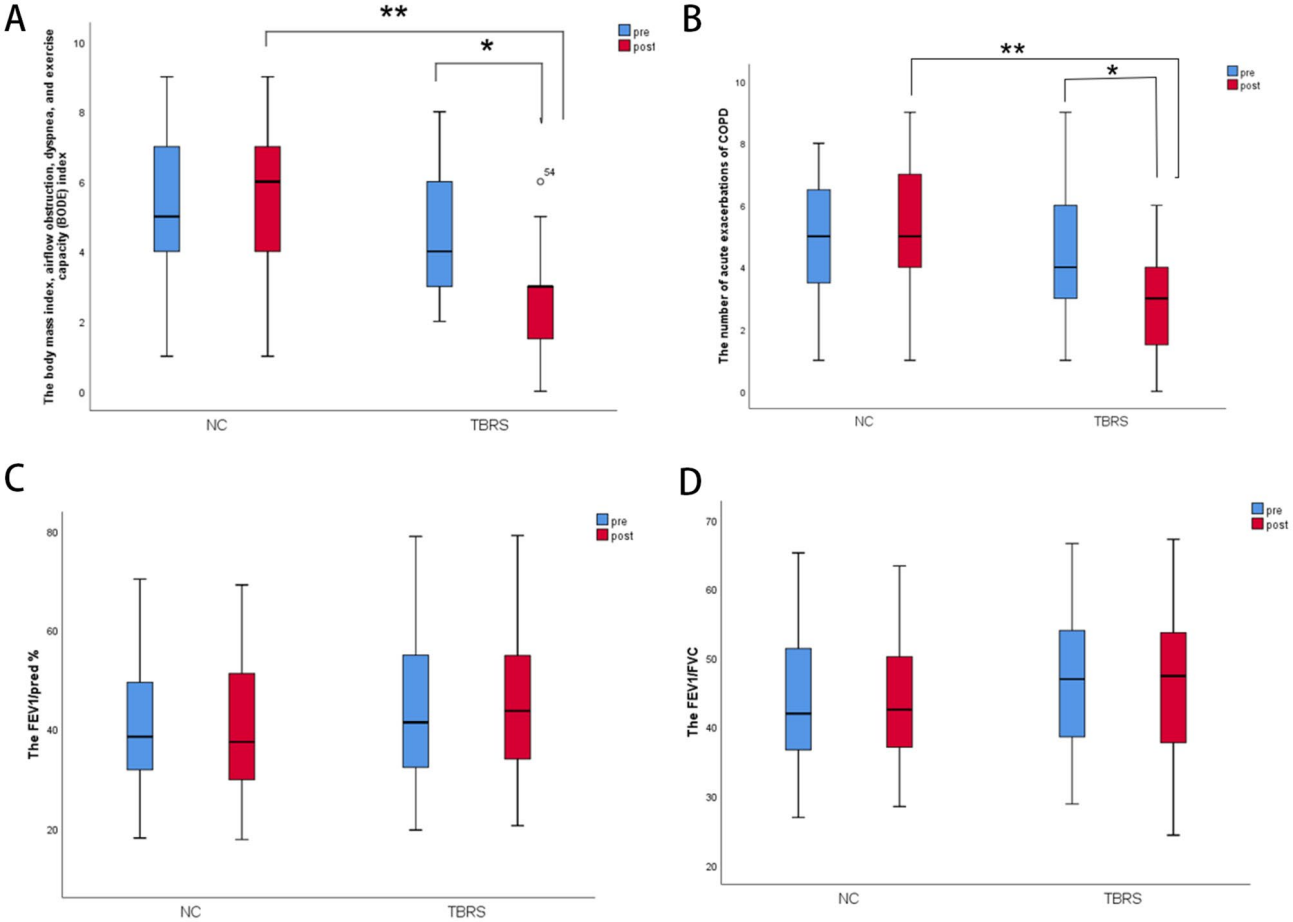
Measurement of pulmonary rehabilitation in the two groups before and after intervention. (**A**) the body mass index, airflow obstruction, dyspnea, and exercise capacity index (BODE); (**B**) the number of acuter exacerbations of COPD; C, FEV_1_/pred%; D, FEV_1_/FVC; (*P = 0.00 compared with before intervention, **P = 0.00 compared with the control group).

After the intervention, compared with before intervention, the CAT score decreased (from 22.15 ± 4.83 to 14.08 ± 3.31, *P* = 0.00), and the BODE index was reduced  in the TBRS group (*P* = 0.00). Compared with the NC group, the CAT score (Fig. 3D) and BODE index (Fig. 4A) was also decreased in the TBRS group (*P* = 0.00). 

#### Assessment of the number of acute exacerbations

After the intervention, compared with before intervention, the number of acute exacerbations of COPD in the past year was reduced in the TBRS group (*P* = 0.00); in a comparison between groups, we found differences between the TBRS group and the NC group (*P* = 0.00) (Fig. 4B).

#### Pulmonary function test

After the intervention, compared with before intervention, there was no significant difference in FEV_1_%pred (Fig. 4C) or FEV_1_/FVC (Fig. 4D) in either the TBRS or NC group. In a comparison between groups, we found no differences.

#### Measurement of oxidative stress

**Figure 5:**
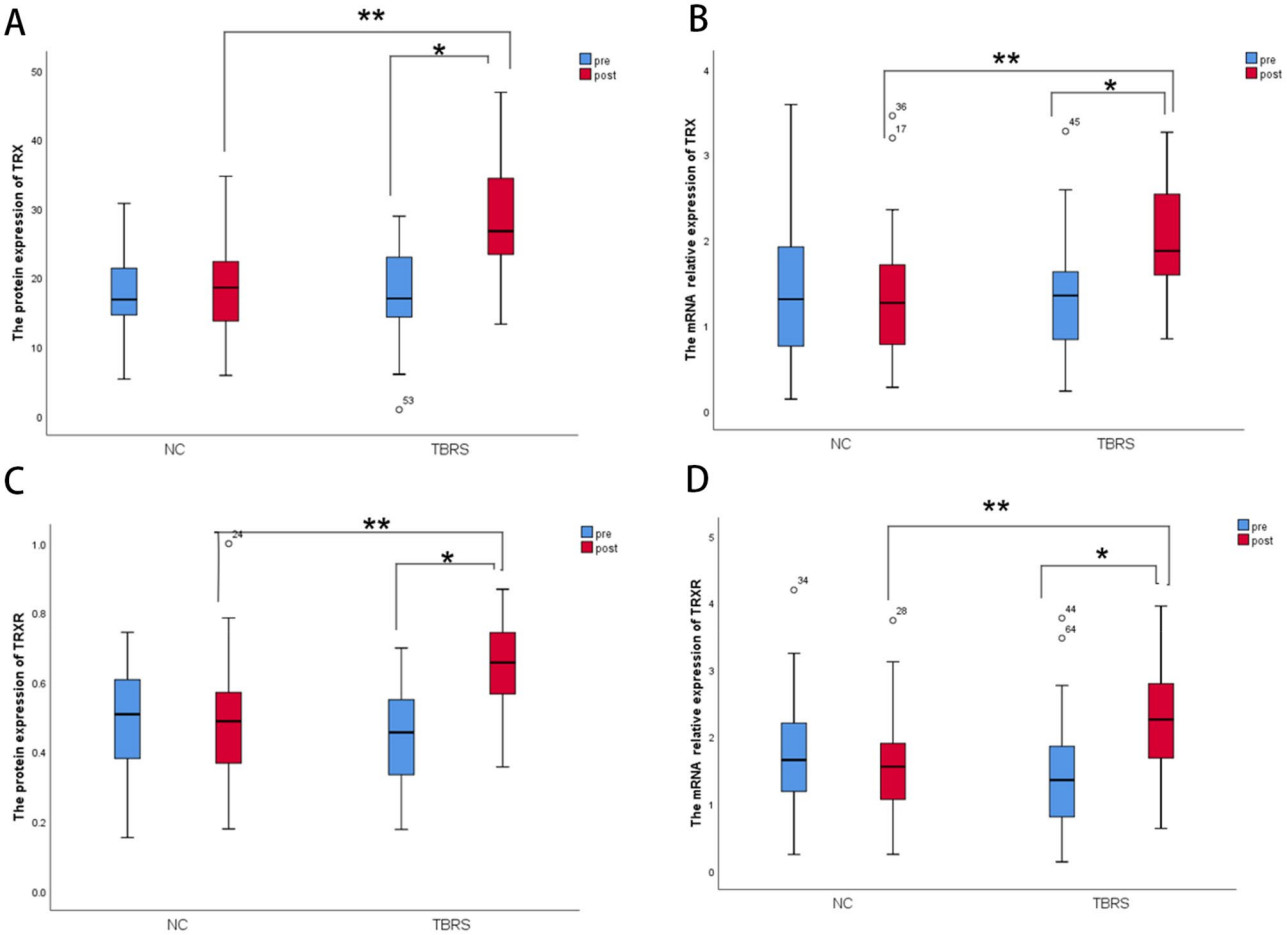
The expressions of TRX, TRXR protein and mRNA in the peripheral blood of the two groups before and after intervention. (**A**) the protein expression of TRX; (**B**) the mRNA relative expression of TRX; (**C**) the protein expression of TRXR; (**D**) the mRNA relative expression of TRXR; (*P = 0.00 compared with before intervention, **P = 0.00 compared with the control group).

After the intervention, compared with before intervention, the protein and mRNA expressions levels of TRX (Fig. 5A,B) and TRXR (Fig. 5C,D) were significantly increased in the TBRS group (*P* = 0.00); in a comparison between groups, the TRX and TRXR protein and mRNA expressions levels in the TBRS group were also higher than those in the NC group (*P* = 0.00).

## Discussion

COPD patients have varying degrees of exercise intolerance, activity limitation and reduced social adaptability, which greatly affect health-related quality of life. This is the first study to explore the effect of TBRS training on exercise capacity and thioredoxin in moderate and severe COPD patients. Our study found that after the intervention, in the TBRS training group, the 6MWD and the protein and mRNA expression levels of TRXS were significantly increased.

Exercise intolerance is one of the major symptoms of COPD, which is affected by multiple factors^29–31^ and greatly affects the health-related quality of life of COPD patients and even leads to inability to carry out activities of daily living and self-care. Muscle disuse will result in essential changes to the peripheral muscles of COPD patients. Systemic inflammation and oxidative stress may further aggravate peripheral muscle dysfunction, thus reducing the exercise tolerance of COPD patients. Studies have shown that oxidative stress induced by a systemic inflammatory response is one of the main mechanisms leading to exercise intolerance. This suggests that alleviating oxidative stress may be a therapeutic target for increasing exercise tolerance in COPD patients^32^. The 6MWD is an independent predictor of exercise capacity^21^ and mortality in the COPD population; the relative mortality risk of COPD is reduced by 0.4% for every metre increase in walking distance, and the mortality risk is reduced by 17.6% for every 50 m increase after pulmonary rehabilitation^32^. Our research found that after the intervention, the 6MWD was significantly improved in the TBRS group (Fig. 3A), which showed that TBRS training significantly improved exercise endurance in moderate and severe COPD patients, consistent with previous studies^2,19,33^.

Activation of the oxidative stress adaptation response is one possible mechanism responsible for the increase in exercise capacity. Oxidative stress is one of the important pathologies of COPD, and TRXS plays a very important role in oxidative stress. In COPD patients, there is a decrease in the function or activity of antioxidant enzymes and the loss or lack of nonenzymatic antioxidants, resulting in oxidation products that cannot be removed or restored in a timely and effective manner and thus an oxidation/antioxidation imbalance^34^. In another study in which needle biopsies of the vastus lateralis before and after endurance training were obtained, COPD patients showed a reduced ability to adapt to endurance training, reflected in a lower capacity to synthesize the antioxidant reduced glutathione^35^.

Although exercise training increases the antioxidant capacity of COPD patients, strenuous exercise and acute exercise loading have the opposite effect, increasing oxidative stress. However, long-term and efficient exercise training efficiently raises antioxidant capacity without increasing oxidative stress^7–9,36,37^. Pinho et al.^9^ found that COPD patients are characterized by increased systemic and pulmonary oxidative stress markers, both at rest and induced by the cardiopulmonary exercise test, and the pulmonary rehabilitation programme was associated with decreased systemic exercise-induced oxidative damage. A study by Mercken et al.^36^ found that COPD patients have increased levels of oxidative stress, and exercise training can effectively reduce the body’s oxidative stress and increase exercise tolerance. Sensson et al.^38^ found that exercise training can enhance the antioxidant activity of glutathione (GSH), which can increase the antioxidant capacity of skeletal muscle and improve exercise endurance. Similarly, Rodriguez et al. reported that in severe COPD patients, high-intensity exercise training of long duration improves exercise capacity while preventing the enhancement of systemic and muscle oxidative stress^39^. Nemoto et al.^40^ conducted 8 weeks of pulmonary rehabilitation exercise training with 12 stable COPD patients. Serum oxidative stress markers were detected before and after the experiment, which showed that exercise training not only improves the exercise capacity of COPD patients but also reduces whole-body oxidative stress.

Our study is the first to analyse the effects of long-term regular exercise training on TRXS. We found that the protein and mRNA expression levels of TRX and TRXR in the serum of COPD patients were increased after 12 weeks of TBRS exercise training (Fig. 5). Therefore, we speculated that long-term regular exercise training may improve the expression of the antioxidant substance TRXS, thus increasing antioxidant capacity and reducing whole-body oxidative stress.

Dyspnoea or exertional dyspnoea is a common clinical symptom in COPD patients, presenting with progressive aggravation and serious effects on health-related quality of life. In our study, the mMRC score was used for preliminary assessment of dyspnoea, and the Borg scale score was mainly used for accurate assessment of the degree of dyspnoea after the 6-min walking test. The results showed that after the intervention, the mMRC score and Borg scale were significantly reduced (Fig. 3B,C), which indicated that TBRS training significantly improved dyspnoea in moderate and severe COPD patients, consistent with previous studies^19,33^.

The BODE index, which includes nutritional status, pulmonary function, exercise status and dyspnoea, comprehensively reflects the systemic status of COPD patients and can predict mortality in COPD patients^41^. Cote et al.^42^ found that after pulmonary rehabilitation, the BODE index improved, and in COPD patients the BODE index improved by 19% but returned to baseline after 2 years, with a mortality rate of 7% after 2 years. In patients who did not receive pulmonary rehabilitation, the BODE index increased by 4% after 1 year and 18% after 2 years, and mortality was as high as 39%. Candemir et al.^43^ found that after pulmonary rehabilitation, changes in BODE scores were significantly correlated with improvements in dyspnoea, exercise capacity and quality of life, and the BODE score could be a better predictor of pulmonary rehabilitation efficacy than some individual variables such as BMI or FEV1.

Our research also found that after 12 weeks of exercise training intervention, TBRS training decreased the CAT score (Fig. 3D) and BODE index (Fig. 4A), and the number of acute exacerbations of COPD in the past year was reduced (Fig. 4B), consistent with previous studies^2,41–43^. However, no differences were found in PF parameters in the comparison before the intervention or between groups (Fig. 4C,D), consistent with previous studies^44,45^.

The principal limitations in this study were that (a) it was a single-centre study with patients from one region; (b) other complications cannot be completely ruled out; (c) no other forms of exercise were included for comparison; (d) other indicators of oxidative stress were not detected and (e) this was a descriptive rather than an in-depth mechanistic study.

## Conclusions

TBRS training can effectively increase exercise capacity, while there are indications that it can alleviate COPD symptoms of dyspnoea and reduce the number of acute exacerbations of COPD. Interesting, long-term regular TBRS training may reduce the oxidative stress associated with COPD to increase exercise capacity.

## Data Availability

The data used and analyzed in this study are available from the corresponding author on reasonable request. E-mail: zhuliming3298@163.com.

## Abbreviations

BMI: Body mass index
6MWD: 6-Minute walk distance
BODE: Body mass index, airflow obstruction, dyspnea, and exercise capacity index
BORG: BORG score
mMRC: Modified British medical research council
CAT: COPD assessment test
PFT: Pulmonary function test
FVC: Forced vital capacity
FEV1: Forced expiratory volume in 1 s
FEV1%pred: Forced expiratory volume in 1 s% prediction
TRX: Thioredoxin
TRXR: Thioredoxin reductase
GSH: Glutathione
TBRS: Total-body recumbent stepper
ELISA: Enzyme-linked immunosorbent assay
Real-time PCR: Real-time Quantitative polymerase chain reaction
mRNA: Messenger ribonucleic acid
EDTA: Ethylene diamietetraacetic acid
HRR: Heart Rate Reserve
THR: Target heart rate
HRmax: Maximum heart rate
HRrest: Rest heart rate
ALS: Amyotrophic Laternal Sclerosis

## References

[1] Lareau SC, Fahy B, Meek P, Wang A (2019). Chronic obstructive pulmonary disease (COPD). Am. J. Respir. Crit. Care Med..

[2] Spruit MA (2013). An official American Thoracic Society/European Respiratory Society statement: Key concepts and advances in pulmonary rehabilitation. Am. J. Resp. Crit. Care Med..

[3] McCulloch J (2018). Prediction of maximal oxygen consumption from rating of perceived exertion (RPE) using a modified total-body recumbent stepper. Int. J. Exer. Sci..

[4] Dalleck LC (2011). Development of a metabolic equation for the NuStep recumbent stepper in older adults. Percept. Mot. Skills.

[5] Billinger SA (2012). Aerobic exercise in subacute stroke improves cardiovascular health and physical performance. J. Neurol. Phys. Ther..

[6] Barnes PJ (2020). Oxidative stress-based therapeutics in COPD. Redox Biol..

[7] Mercken EM (2009). Systemic and pulmonary oxidative stress after single-leg exercise in COPD. Chest.

[8] Ryrsø CK (2018). Effect of endurance versus resistance training on local muscle and systemic inflammation and oxidative stress in COPD. Scand. J. Med. Sci. Sports.

[9] Pinho RA (2007). Oxidative stress in chronic obstructive pulmonary disease patients submitted to a rehabilitation program. Respir. Med..

[10] García-Rio F (2011). Dynamic hyperinflation, arterial blood oxygen, and airway oxidative stress in stable patients with COPD. Chest.

[11] Stanojkovic I (2011). Pulmonary function, oxidative stress and inflammatory markers in moderate and severe COPD exacerbation. Respir. Med..

[12] Matsuo Y, Yodoi J (2013). Extracellular thioredoxin: A therapeutic tool to combat inflammation. Cytokine Growth Factor Rev..

[13] Rhee SG (2005). Peroxiredoxins: A historical overview and speculative preview of novel mechanisms and emerging concepts in cell signaling. Free Radic. Biol. Med..

[14] Kraemer WJ, Ratamess NA (2005). Hormonal responses and adaptations to resistance exercise and training. Sports Med..

[15] Moher D, Hopewell S, Schulz KF (2012). CONSORT 2010 explanation and elaboration: Updated guidelines for reporting parallel group randomised trials. Int. J. Surg..

[16] Global Initiative for Chronic Obstructive Lung Disease. Global strategy for the diagnosis, management, and prevention of chronic obstructive pulmonary disease revised 2016 [OL]. http://www.Gold--copd.org/guidelines-global-strategy-for-diagnosis-management.Html (2016).

[17] Dayan F (2006). The oxygen sensor factor-inhibiting hypoxia-inducible factor-1 controls expression of distinct genes through the bifunctional transcriptional character of hypoxia-inducible factor-1alpha. Cancer Res..

[18] Myers CR, Myers JM (2009). The effects of acrolein on peroxiredoxins, thioredoxins, and thioredoxin reductase in human bronchial epithelial cells. Toxicology.

[19] Kawagoshi A (2015). Effects of low-intensity exercise and home-based pulmonary rehabilitation with pedometer feedback on physical activity in elderly patients with chronic obstructive pulmonary disease. Respir. Med..

[20] Thompson PD (2013). ACSM's new preparticipation health screening recommendations from ACSM's guidelines for exercise testing and prescription, ninth edition. Curr. Sports Med. Rep..

[21] ATS Committee on Proficiency Standards for Clinical Pulmonary Function Laboratories (2002). ATS statement: Guidelines for the six-minute walk test. Am. J. Respir. Crit. Care Med..

[22] Sweer L, Zwillich CW (1990). Dyspnea in the patient with chronic obstructive pulmonary disease. Etiology and management. Clin. Chest Med..

[23] Borg GA (1982). Psychophysical bases of perceived exertion. Med. Sci. Sports Exerc..

[24] Jones PW (2009). Development and first validation of the COPD Assessment Test. Eur. Respir. J..

[25] Celli BR (2004). The body-mass index, airflow obstruction, dyspnoea, and exercise capacity index in chronic obstructive pulmonary disease. N. Engl. J. Med..

[26] Global Initiative for Chronic Lung Disease. *GOLD Spirometry Guide—2010 Update* (accessed November 2, 2011). http://www.goldcopd.org/other-resources-gold-spirometry-guide.html.

[27] Barakat S, Michele G, George P, Nicole V, Guy A (2008). Outpatient pulmonary rehabilitation in patients with chronic obstructive pulmonary disease. Int. J. Chron. Obstruct. Pulmon. Dis..

[28] Spruit MA (2012). Age-graded reductions in quadriceps muscle strength and peak aerobic capacity in COPD. Revista brasileira de fisioterapia (Sao Carlos (Sao Paulo, Brazil).

[29] Aliverti A, Macklem PT (2008). The major limitation to exercise performance in COPD is inadequate energy supply to the respiratory and locomotor muscles. J. Appl. Physiol..

[30] Debigaré R, Maltais F (2008). The major limitation to exercise performance in COPD is lower limb muscle dysfunction. J. Appl. Physiol..

[31] Foschino Barbaro MP (2007). Inflammation, oxidative stress and systemic effects in mild chronic obstructive pulmonary disease. Int. J. Immunopathol. Pharmacol..

[32] Houchen-Wolloff L (2017). Survival following pulmonary rehabilitation in patients with COPD: the effect of program completion and change in incremental shuttle walking test distance. Int. J. Chron. Obstruct. Pulmon. Dis..

[33] Covey MK (2014). Resistance training as a preconditioning strategy for enhancing aerobic exercise training outcomes in COPD. Respir. Med..

[34] Cornwell WD (2010). Pathogenesis of inflammation and repair in advanced COPD. Semin. Respir. Crit. Care Med..

[35] Rabinovich RA (2001). Reduced muscle redox capacity after endurance training in patients with chronic obstructive pulmonary disease. Am. J. Respir. Crit. Care Med..

[36] Mercken EM (2005). Rehabilitation decreases exercise-induced oxidative stress in chronic obstructive pulmonary disease. Am. J. Respir. Crit. Care Med..

[37] Oh-ishi S (1997). Endurance training improves the resistance of rat diaphragm to exercise-induced oxidative stress. Am. J. Respir. Crit. Care Med..

[38] Svensson MB (2002). Adaptive stress response of glutathione and uric acid metabolism in man following controlled exercise and diet. Acta Physiol. Scand..

[39] Rodriguez DA (2012). Muscle and blood redox status after exercise training in severe COPD patients. Free Radic. Biol. Med..

[40] Nemoto K, Itoh M, Nakamura H (2012). Effect of exercise therapy on reactive oxygen species and reactive nitrogen species in COPD patients. J. Tokyo Med. Univ..

[41] Cote CG (2008). The modified BODE index: validation with mortality in COPD. Eur. Respir. J..

[42] Cote CG, Celli BR (2005). Pulmonary rehabilitation and the BODE index in COPD. Eur. Respir. J..

[43] Candemir İ (2019). Use of i-BODE index to determine efficacy of pulmonary rehabilitation in COPD patients. Tuberkuloz ve toraks..

[44] Franssen FM, Broekhuizen R, Janssen PP (2004). Effects of whole-body exercise training on body composition and functional capacity in normal-weight patients with COPD. Chest.

[45] Spruit MA, Gosselink R, Troosters T (2002). Resistance versus endurance training in patients with COPD and peripheral muscle weakness. Eur. Respir. J..

